# Pediatricians’ experiences of managing outpatient care during the COVID-19 pandemic: A qualitative study in Germany

**DOI:** 10.3389/fped.2023.1127238

**Published:** 2023-04-17

**Authors:** Julia von Sommoggy, Eva-Maria Grepmeier, Christian Apfelbacher, Susanne Brandstetter, Janina Curbach

**Affiliations:** ^1^ Medical Sociology, Institute of Epidemiology and Preventive Medicine, University of Regensburg, Regensburg, Germany; ^2^ Institute of Social Medicine and Health Systems Research, Otto von Guericke University Magdeburg, Medical Faculty, Magdeburg, Germany; ^3^ Family Medicine and Primary Care, Lee Kong Chian School of Medicine, Nanyang Technological University, Singapore, Singapore; ^4^ University Children's Hospital Regensburg (KUNO), University of Regensburg, Klinik St. Hedwig, Regensburg, Germany; ^5^ Department of Business Studies, Ostbayerische Technische Hochschule Regensburg, Regensburg, Germany

**Keywords:** COVID-19, pediatric care, outpatient care, content analysis, pandemic

## Abstract

**Background:**

Pediatricians are important sources of information for parents regarding their children's health. During the COVID-19 pandemic, pediatricians faced a variety of challenges regarding information uptake and transfer to patients, practice organization and consultations for families. This qualitative study aimed at shedding light on German pediatricians’ experiences of providing outpatient care during the first year of the pandemic.

**Methods:**

We conducted 19 semi-structured, in-depth interviews with pediatricians in Germany from July 2020 to February 2021. All interviews were audio recorded, transcribed, pseudonymized, coded, and subjected to content analysis.

**Results:**

Pediatricians felt able to keep up to date regarding COVID-19 regulations. However, staying informed was time consuming and onerous. Informing the patients was perceived as strenuous, especially when political decisions had not been officially communicated to pediatricians or if the recommendations were not supported by the professional judgment of the interviewees. Some felt that they were not taken seriously or adequately involved in political decisions. Parents were reported to consider pediatric practices as sources of information also for non-medical inquiries. Answering these questions was time consuming for the practice personnel and involved non-billable hours. Practices had to adapt their set-up and organization immediately to the new circumstances of the pandemic, which proved costly and laborious as well. Some changes in the organization of routine care, such as the separation of appointments for patients with acute infection from preventive appointments, were perceived as positive and effective by some study participants. Telephone and online consultations were established at the beginning of the pandemic and considered helpful for some situations, whereas for others these methods were deemed insufficient (e.g. for examinations of sick children). All pediatricians reported reduced utilization mainly due to a decline in acute infections. However, preventive medical check-ups and immunization appointments were reported to be mostly attended.

**Conclusion:**

Positive experiences of reorganizing pediatric practice should be disseminated as “best practices” in order to improve future pediatric health services. Further research could show how some of these positive experiences in reorganizing care during the pandemic are to be maintained by pediatricians in the future.

## Introduction

The COVID-19 pandemic put great strains on health care professionals (HCPs) and health systems. Even though the clinical course of the COVID-19 disease is milder and prognosis is better for children than adults (1), children face specific risks, e.g., the pediatric inflammatory multisystem syndrome, which occurred in a small, but still significant number of children with a COVID-19 infection (2–4) and long-term complications are not foreseeable (5). Furthermore children, especially infants, are vulnerable and thus in close contact with pediatricians especially during their first year of life, e.g., taking up routine clinical examinations or vaccinations (6). In Germany, a great majority (90%) of the children <6 years of age is seen by a pediatrician (typically working in private practice), who is specifically prepared with a 5-year period of postgraduate pediatric training in certified hospitals and outpatient institutes. Thirteen routine clinical examinations are offered to children from birth to adolescence (7). Thus, the position of pediatricians providing outpatient care is unique within the German healthcare system, even more so during a pandemic.

During the course of the first year of the pandemic (2020), the majority of the German general population felt well informed about COVID-19 and displayed high levels of evidence-based knowledge, as a lot of information was available (8, 9). However, over half of the population at the same time felt confused due to the dramatic increase in availability of scientific evidence mixed with false information and was uncertain which information sources to trust (10). The World Health Organization (WHO) declared this an “infodemic” (11). It is a task of pediatricians to convey health-related information to parents in an understandable way, enabling them to act accordingly (12, 13). Thus, pediatricians had to stay up to date on the latest scientific evidence in order to be able to provide comprehensive care for their patients. Over the course of the pandemic, new information emerged that was relevant for parents, e.g., regarding infection protection measures, testing, vaccination of children, high infection rates within childcare institutions/schools (14–18). The basis for health-related decisions constantly changed, which sometimes made it difficult to stay informed even for medical experts (9, 19, 11, 20).

While pediatricians had to adapt to providing advice on COVID-19-related child health during the pandemic, pediatric encounters were also affected by hygiene and infection protection regulations. Pediatricians who provide outpatient care had to make considerable adjustments to the way their practice was set up in order to continue patient care (including testing for COVID-19) and consultations (including discussing COVID-19-related issues). To ensure safe patient care, they were required to implement hygiene and infection prevention/control measures in their practice (e.g., sneeze guards, wearing facial masks) and procedures (e.g., separation of sick and healthy patients) and communicate these to their patients (21, 22). Wearing face masks influenced pediatric encounters significantly, as it interrupted the ability of pediatricians to interact with the children (23, 22).

Moreover, HCPs had to deal with varying utilization of appointments over the course of the pandemic (24). Whereas patients with symptoms suggesting a COVID-19 infection had been advised not to visit medical practices in person, many patients with other serious symptoms were also afraid to visit their doctor, or did not attend preventive medical check-ups for fear of becoming infected with COVID-19 (25, 26). In pediatric practices emergency visits were significantly reduced, whereas developmental examinations remained mostly unchanged, compared to the previous year (27). Studies show, that vaccination uptake was affected during the pandemic, however could be resumed (28, 29). Additionally comprehensive provision of care was ensured also by introducing remote consultation via telephone/video during acute phases, e.g., lockdowns (20, 30).

In order to build resilient health systems, it is necessary to understand challenges and gain a deeper understanding how to cope with them (31). Several studies focus on experiences in primary and hospital care during the COVID-19 pandemic (32, 33). Pediatric outpatient care has specific challenges, e.g., providing care for very young patients who are not able to communicate with words, and it is thus necessary to specifically focus on this group of health care providers. Therefore, the aim of this study was to shed light on the challenges experienced by German pediatricians in providing outpatient care during the first year of the COVID-19 pandemic.

## Methods

### Study design

An explorative qualitative study design was chosen in order to capture diverse perspectives and to gain a broad insight into the experiences of pediatricians in their daily professional lives during the pandemic. Qualitative research allows to understand social phenomena in depth, while statistical representativeness is not aimed for (34). This approach has the potential to capture themes and topics that might not emerge when working with predefined, standardized categories and assumptions (35).

### Data collection

Two experienced researchers working as a research assistant and a research fellow in the Department of Medical Sociology at the University of Regensburg (EMG, M.A. Comparative European Ethnology and JvS, PHD Comparative European Ethnology) conducted semi-structured interviews (*n* = 19) via telephone from July 2020 to February 2021. We opted for semi-structured interviews to enable us to maintain a focus on certain topics, while remaining open to aspects spontaneously arising during the interviews.

To gain insight into changes to daily professional practice of pediatricians induced by the COVID-19 pandemic, the interview guide (cf. Supplementary Data Sheet S1) comprised questions regarding a) general changes in routine work as perceived by the study participants, b) information acquisition by study participants regarding COVID-19 health-related topics c) information transfer to and consultation of parents regarding COVID-19-related health information.

To create an atmosphere of openness, the pediatricians were encouraged to speak freely and share their thoughts on their experiences in the beginning of the interviews. This was followed by more structured questions of the interview guide. Topics that seemed highly relevant to any given interviewee were discussed in depth. Most pediatricians gave positive feedback after the interview, stating that they perceived the atmosphere of the conversation to be very relaxed and characterized by openness. Some interviewees were restricted by upcoming appointments and may thus have been slightly stressed. Participants received an incentive of €50 and participated voluntarily.

The reporting of this study adheres to the COREQ standard for reporting qualitative research results (36).

### Recruitment

A purposive sampling strategy was used by recruiting single and shared practices from urban and rural areas. We expected patients to be more diverse in terms of socio-economic status, ethnicity, living conditions, information needs and ability to adhere to social distancing in urban compared to rural areas. Working in an individual or a shared practice might have an impact on the uptake of information and also on the safety and infection protection measures undertaken. By recruiting specific groups of pediatricians, who possess certain characteristics relevant to the topic being studied, we aimed for a maximum variation of perspectives (34, 37).

To recruit interviewees, initial contact was established with a Bavarian association of pediatricians “PaedNetz Bayern” and with the North Rhine Professional Organization of Pediatricians. With the help of these organizations, interview partners from Bavaria (southern Germany) and North Rhine-Westphalia (mid/northern Germany) were recruited. Subsequent snowballing and personal contacts completed our sample. There was no relationship established between the researchers and the interviewees prior to the interviews and besides information on the study background and scope no further information was provided beforehand. Recruitment was stopped on reaching theoretical saturation (when no new themes came up during the interviews). After ten interviews, the two researchers conducting the interviews (JvS and EG) began to discuss topics and themes in the data to establish whether data saturation had been reached. They agreed that after 15 interviews no new topics or themes had emerged. Four more interviews were conducted to ensure theoretical saturation (38).

### Sample

The interviews took place between July 2020 and February 2021. Nine study participants were male, ten female. They worked in North Rhine-Westphalia (*n* = 9) or Bavaria (*n* = 10), respectively, with an age range between 39 and 67 years. Eight pediatricians of our sample worked in an individual practice, whereas eleven worked in a shared practice. Concerning the catchment areas, six interviewees worked in villages/small towns (<20,000 inhabitants), 13 worked in a medium-sized to large town (>20,000 inhabitants) (cf. Table 1).

**Table 1:** Pediatricians included in our study.

	Pediatricians (*n* = 19)
**Catchment area**	
Village/small town (<20,000 inhabitants (IN))	6
Medium-sized/large town (>20,000 IN)	13
**Gender**	
Male	9
Female	10
**Practice**	
Individual practice	8
Shared practice	11
**Age**	
>50 years of age	11
<50 years of age	8
**Experience**	
>15 years of experience	11
<15 years of experience	8

### Analysis

The total length of the interviews, which included one further topic, namely health literacy in allergy prevention, was 26–103 min (mean: 48 min). About half the time of the interviews, the focus was on COVID-19 (mean: 24 min). All interviews were conducted by telephone, audio recorded, transcribed verbatim and pseudonymized. Initially, three interviews were discussed and coded jointly by three researchers (JvS, EG, JC) using ATLAS.ti (version 8). JC is principal investigator of the project and senior researcher working as research fellow in the Department of Medical Sociology at the University of Regensburg (PHD political science). Codes were developed deductively, based on the interview guide, and inductively derived from emerging themes in the interview data. Once codes were defined, all interviews were coded independently by the researchers using the jointly developed codes. Every interview was coded by two researchers, subsequently codes were compared, and differences discussed until consensus was reached. In addition “deviant” views were examined with specific thoroughness to ensure rigor (39). This was followed by content analysis (JvS, EG, JC) (40, 41). By grouping codes into higher level themes, overarching topics were identified and summarized (42).

### Informed consent, confidentiality and ethics approval

The study received ethical approval from the Ethics Committee of the University of Regensburg (18–1205–101). Participation in the study was only possible after providing informed consent to the audio recording and scientific use of the interview data. Data storage and handling of personal information follows the data protection policy of the Medical Sociology of the University of Regensburg (43).

## Results

The interviews revealed that the pediatricians were confronted with four main challenges (cf. Table 2) during the pandemic.

**Table 2:** Overview over the main challenges for pediatricians during the COVID-19 pandemic.

Challenge 1: Information acquisition and advice on COVID-19 by pediatricians	Information overload due to high media coverage	
	High demand on keeping up-to-date for pediatricians	
	Organizational challenges within the practices due to lack of information, e.g., regarding accounting regulations	
	Consultations on non-medical issues, e.g., on administrative questions, testing strategies, quarantine regulations	
	Perceived lack of considering pediatric expertise in political decisions	
Challenge 2: Infection protection—reorganizing the practice	(Costly) changes in practice set up, e.g., installation of sneeze-guards	
	Separation of patients with acute infections and non-infectious patients	
	+ Positive appraisal: Perceived as advantageous for organizing practice	- Negative appraisal: Financial burden due to reduced patient numbers
	Coping with staff shortage due to infections and quarantine regulations	
Challenge 3: Consulting—while keeping a distance	Introduction of telephone/online consultations in pediatric practices	
	+ Positive appraisal: Useful for some medical questions	- Negative appraisal: Physical examination of sick children was considered essential
	Hampered patient consultations due to face masks, especially with young children	
Challenge 4: Changes in disease pattern and health service utilization	Drop in patient numbers due to patients’ fear of infection and reduced number acute mild infections in children	
	+ Positive appraisal: Reduced workload was perceived as relief for some practices	- Negative appraisal: drop in utilization posed economic difficulties for others
	Routine check-ups were conducted continuously or caught up to	
	Vaccinations were sometimes easier to administer due to reduced infection rates in children	
	Some practices made efforts to teach parents in treating mild infection themselves at home	

### Challenge 1: Information acquisition and advice on COVID-19

#### Information acquisition and staying up-to-date of pediatricians

The interviewees reported that they had to keep up to date about changes regarding COVID-19-related regulations and recommendations. All pediatricians stated that they generally felt capable of keeping informed on the pandemic and that they used various trustworthy official sources to obtain the necessary information. However, as the amount of COVID-19-related information and recommendations was sometimes overwhelming, it was important and helpful to exchange views and communicate with colleagues and the practice team in order to affirm the professional position.

*And then, every other week, we sit down as a team and discuss how do we approach this? What is our position? How do our employees communicate this to patients on the phone? (Int 18)*

Keeping up to date was reported as being very time consuming and representing an additional burden. Obtaining information regarding regulations on mandatory infection protection measures within the practice setting and pandemic-related administrative and accounting challenges was reported as being particularly strenuous. For example, one of the challenges mentioned by the interviewees was to find out how to bill an infant check-up visit which is usually scheduled in advance and has to be performed at a certain age but had to be postponed due to social distancing recommendations. The pediatricians reported they were frequently unclear about how to solve these non-routine administrative tasks and information was difficult to obtain.

*For more than a year now, every two, three or four weeks, we've been struggling with uncertainties, on which we have to work out our own personal position. How do we actually handle this now? It's painstaking. […] And there are so many administrative rather than medical questions. (Int 18)*

*I think for all of us these everchanging rules regarding billing are extremely stressful. And it is also disrupting our daily professional working life, which is a huge stress factor. (Int 15)*

#### Consultations beyond medical issues

At the same time, it is not only information regarding practice organization and administration that was frequently changing, but also regulations concerning families’ lives, e.g., closure and reopening of childcare and schools, or obligation of school children to wear masks and get tested on a regular basis. The interviewees reported that, despite not being an exclusively medical issue, the rapidly changing and sometimes unclear recommendations and rules pertaining to family life were a major concern for parents. Hence, the pediatricians were frequently asked how to handle these topics. The interviewees also reported that due to the high level of media coverage, they sometimes received COVID-19-related information from parents before political decisions could actually be implemented and officially conveyed to them as physicians. This lack of (or delay in) official, written communication made it challenging for the health professionals to provide parents with the necessary care and medical documents, e.g., COVID-19 tests or medical certificates needed for children to attend school.

*Parents came and told us that the health department had said so….* *But of course, we can’t rely on things we hear from parents. You need at least some kind of documentation, like an e-mail, phone call or written document to confirm the origin of these instructions. (Int 12)*

#### Lack of consideration of pediatricians’ expertise on a political level

Some pediatricians in our sample felt that political decisions on children's health and well-being were rushed into without considering pediatric expertise or pediatricians’ professional scope of action in the healthcare system. They felt that they had no voice in the political decision-making process and that their involvement in implementing these decisions was detached from the political arena.

*You virtually can't provide proper medical care and it also became evident that the expertise of physicians and pediatricians is not considered by policymakers or the administration when designing hygiene plans etc. Medicine and epidemiology are carried out by lawyers who, to put it bluntly, are not exactly the most knowledgeable in medical issues. (Int 7)*

Accordingly, some of the pediatricians interviewed were critical of the fact that they had to explain COVID-19-related political decisions and regulations that they did not understand or support themselves, which made it even more challenging to communicate and explain these to parents:

*It was difficult to communicate a testing strategy when it wasn’t even clear to me. (Int 14)*

*And sometimes we also have the problem that politicians say: Yes, you can get that from your doctor. But we have to say: We are not allowed to issue this to you. That's exactly what happened with the flu vaccination in the autumn. The German Health Minister said: All children and adolescents will now be vaccinated against influenza. But we had to tell the parents that it's nice he told them that, but their insurance won't pay for it. (Int 11)*

*I can’t certify “this child is healthy” for daycare because I can’t 100 percent rule out a corona infection. This is a difficult situation for us. Everyone tries to sort of pass the buck, and at the end of the chain there is the pediatrician. (Int 5)*

#### Increased workload due to providing health information to parents

In order to provide patients with COVID-19-related information, the pediatricians reported using different strategies and information channels. Many reported posting information on a website, Facebook site or sending it to parents via e-mail. Physician assistants were assigned the task of passing on information regarding practice organization and infection protection measures to patients. A lot of pediatricians reported a high workload for receptionists as they were frequently contacted by uncertain parents asking them legal questions on how to proceed when their children had been identified as contact persons or tested positive for COVID-19.

*Sometimes people can't reach us by phone. The problem is that the health authorities are overburdened. This means: a mother calls and says: “My daughter*'*s friend tested positive, and they were in contact yesterday. But I can't reach anyone at the health department. What do I have to do now?” I get a lot of these questions — and also trivial legal questions. (Int 11)*

### Challenge 2: Infection protection — reorganizing the practice

#### Reorganization of practice set-up

All pediatricians reported having implemented a range of COVID-19 infection protection measures in their practice setting and organization, most even before changes were mandatory by law. Some of these changes were physical changes within the practice setting, e.g., the use of screens as “sneeze guards”, removal of toys from the waiting rooms. The installation of screens, in particular, proved to be costly.

#### Reorganization of appointments

Other changes related to contact between patients and personnel. Use of hand sanitizer was enhanced, social distancing measures were introduced and personal protective equipment (PPE), such as masks, became obligatory, which was exhausting and unpleasant for personnel (especially examining infected children in full PPE). Some pediatricians even closed their waiting rooms and asked parents and children to wait outside or come exactly on time for their appointment. Especially when it came to COVID-19 tests with potentially infectious children, creative alternatives were established by some practices, e.g., performing the tests in the carpark.

*In addition, we also offer the corona swabs in the carpark. Of course, this is very different. I've never examined a child in the carpark before. Yes, this has changed massively. (Int 18)*

Some changes were perceived positively by some, whereas for others this posed an additional burden, e.g., the separation of patients with acute infections and non-infectious patients for medical check-ups. Some pediatricians reported these newly introduced changes in practice organization to be more efficient for their care work and practice procedures. Others claimed that this constant reorganization placed additional stress on staff and the practice had to deal with financial burdens, too.

*I will definitely continue to separate infectious and non-infectious patients after the pandemic, because it helps me to structure my work much better. (Int 8)*

*We are working much less efficiently in economic terms because we cannot squeeze in an appointment with a child with a cough quickly between two routine check-up appointments. If the screening patient doesn’t show up, we are sitting around for 30 min without a patient. (Int 11)*

#### Staff shortage

Furthermore, some practices, especially small ones, had difficulties continuing to operate due to staff shortages caused by COVID-19 infections or quarantine of staff. This placed additional financial stress on the practices.

*Constant reorganization, existential hardships in fact. We’ve had corona cases in our practice and thus also personnel shortages. (Int 12)*

### Challenge 3: Consulting—while keeping a distance

#### Introduction of telephone consultations

Infection protection measures and uncertainty among pediatricians and parents also led to changes in how patient consultations were conducted. The pediatricians reported that at the beginning of the pandemic, there was an increase in telephone consultations, especially questions regarding children's behavior, development, and learning, which did not necessarily require a physical, face-to-face examination. Even after the first lockdown and when infection rates dropped, telephone consultations continued and some practices started using online tools to organize the practice.

*A lot of consultations can be conducted by telephone. Now it is more acceptable for the patients not to come to the practice to be examined. […] And we also use Doctolib, the online appointment system. (Int 16)*

However, most pediatricians emphasized that personal contact is mandatory when it comes to the physical examination of children who are showing symptoms. The physicians expressed the importance of physical contact with children in order to properly diagnose their condition and needs.

*I still can't examine children without touching them. No matter what they say — I can't examine a child at a distance. This isn’t possible, which is why they are treated in the normal way. (Int 4)*

#### Introduction of online consultations

Online consultation was established in just a few practices. Most pediatricians reported not feeling comfortable providing advice online without actually seeing a sick child. The same applied to the parents. Pediatricians reported that parents seemed to prefer to come to the practice rather than have an online consultation when their child was unwell.

*We offer video consultation hours. During the first wave of the pandemic, we had a lot of video consultations. But I have the impression that many people are happy to come to the practice when their child is seriously ill. […] We try to answer pure information inquiries via video consultation, for example: “Is my child allowed to take part in physical education without a mask or not?” or “My child has a heart defect? Can he go to school, or not?” These are questions for which no physical examination is necessary. (Int 10)*

#### Hampered patient consultations due to facial masks

When it comes to conducting patient consultations vis-a-vis in the practice, the interviewees reported that examining children while wearing a face mask was especially challenging with toddlers and infants, as these are often afraid of the examination itself and even more so with a health professional wearing a mask. Wearing a mask is reported as disadvantageous with older children as well when consulting in cases of psychosomatic conditions. The lack of facial expressions, on both sides, makes it difficult to connect with the patient.

*You have to laugh with your eyes. Sometimes it's difficult, and the kids are a bit scared. What I find particularly negative are conversations on psychosomatic issues, where you also have to perceive the reaction of the other person during conversation. Did you poke somewhere and trigger a reaction, is there something, did you hit something? Did you go too far, did you hurt someone, or is he mocking the question because it's not relevant? All of this is impacted by mouth and face coverings. I have to say that conducting conversations with a pubescent patient who has strong psychosomatic problems is quite difficult. (Int 7)*

### Challenge 4: Changes in disease pattern and health services utilization by pediatric patients

#### Drop of patient numbers in the beginning of the pandemic

All pediatricians stated that during the course of the first months of the pandemic and the following lockdowns, there was a massive drop in patient numbers with mild infections. Infections were reduced by infection protection measures, such as hand hygiene, wearing face masks, reduced social contact and the closure of childcare facilities and schools. Some pediatricians suspected a decline in utilization might also have been associated with parents’ fear of visiting a practice and risking an infection. Additionally, a lot of parents worked at home due to the pandemic, and so did not need a medical attestation for their children and were able to take care of the child at home themselves.

*For example, on a Monday, like today, my colleague and I normally see about a hundred patients. At the moment we are seeing about forty. Sometimes only thirty. (Int 18)*

#### Continuous conduction of routine check-ups

All pediatricians recounted that routine check-ups for children were conducted almost continuously, even during lockdowns, and especially for the younger children. Some interviewees reported having restricted the preventive medical check-ups in the first few months of the pandemic to infants under the age of six months and postponing the check-ups for older children. However, the practices caught up with most of their check-ups over the course of 2020. These were sometimes the main business of pediatric practices.

*During lockdown and at times when there were fewer acute cases, we've been able to carry out more preventive check-ups, and that's straightened out our schedule a bit. (Int 5)*

#### Adherence to routine vaccinations

Routine vaccinations were perceived to be easier to administer during the pandemic than in normal times, as all the children receiving vaccines were healthy, because of contact restrictions among others, and immunizations thus did not have to be postponed due to mild infections.

#### Teaching parents on self-management of health issues

Some pediatricians reported that at the beginning of the pandemic, they tried to guide and teach parents to be able to assess the severity of their children's illnesses. The aim was to make them feel more confident about treating mild infections themselves in order to reduce the number of patients visiting the practice:

*At the beginning of the pandemic, we provided patients with a lot of guidance. We changed the website. We sent out mails. And we educated parents on what normal infections actually are, what normal infection processes are, and when they really need to go to the doctor. We also actively promoted the new “FeverApp” developed by the University of Witten/Herdecke. I believe that this has enabled us to train parents even better so that they can deal with mild infections more effectively. (Int 9)*

#### Perspectives on changes in health services utilization

The study participants had different perspectives on the pandemic-related changes in utilization: Some appreciated the reduced number of patients and the fact that patient visits were restricted to more severe cases of “really sick” children:

*In the past, we often asked ourselves, why were the last three patients actually here? And of course, as a pediatrician, you say its better they come once too often, because parents sometimes can't assess the situation correctly. But I have the feeling that the really sick ones are coming to the practice and the others are perhaps staying at home, which I don't think is bad. (Int 9)*

*And it was nice, all of these odds and ends … which are so annoying: A child who has had a runny nose for ten minutes, a child who had coughed or had diarrhea twice and all this nonsense, it all stopped. It was two months of true relief for us. (Int 4)*

Whereas for others this drop in patient numbers posed difficulties, especially regarding the practice's financial situation.

*Hardly any sick patients show up any more. …and this decline is a lot, especially if you have to keep a practice going, with rent, staff, etc. (Int 12)*

## Discussion

In our study, we were able to gain insight into the changes and challenges in the routine care provided by German pediatricians during the first year of the COVID-19 pandemic. Generally, the interviewees in our sample felt able to keep themselves informed on COVID-19, infection prevention measures, regulations regarding COVID-19 and to convey COVID-19-related information to parents. However, they perceived this additional workload as a major burden as the high amount of COVID-19-related information and rapidly changing political recommendations required them to engage actively in time-consuming information retrieval and exchange with colleagues, in establishing new information channels and in extended consultations with parents. Most of these information-related tasks did not revolve around medical information on COVID-19 itself, but on how to implement political recommendations and how to deal with administrative challenges in family life and in the medical practice (setting). The physicians felt that political decision-making was detached from what they and their patients could realistically implement in their routine work as physicians and in family life, and moreover they felt as though they were not taken seriously by politicians and were insufficiently involved in political decisions. This feeling of being insufficiently involved in political decision-making and information flow became manifest for some pediatricians when they received the latest news on COVID-19 regulations from their patients instead of being updated via other professional channels.

Practices had to adjust to infection protection measures, appointments had to be restructured and all this had to be communicated to parents. Most of these changes posed an additional financial burden for the practice, but also placed pressure on staff. Some changes proved to be beneficial for some, e.g., the separation of appointments for acute infections from those focused on preventive measures may be kept on after the pandemic. Telephone and online consultations increased but were also perceived ambiguously. For some, this seems to be an option for the future when consulting on preventive measures, but generally the physical examination of children seems to be mandatory for pediatricians and parents alike, especially when children are in physical distress.

In a study on COVID-19 and pediatric health services in Australia and New Zealand with 468 participating physicians, 79.9% reported having good knowledge of where to find local information on how to manage patients. The majority felt well informed about current pediatric testing (71.1%) and infection control (75.2%) (44). This is in line with our findings, with all the pediatricians in our smaller, qualitative sample stating that they felt well informed, or at least able to access the required information, even though it was sometimes rather overwhelming, difficult to sort through and assess. As in our study, participants in another qualitative study on outpatient care in rural Germany describe the information exchange between colleagues in the management of pandemic information as important and helpful (45). Similarly, a quantitative study on the challenges of the first months of the pandemic in Germany showed that the workload of general practitioners (GPs) was highly increased because information had to be retrieved and corresponding measures implemented on a daily basis (20). These GPs, as well as the interviewees in our study, claimed that their professional experience was not considered in political planning processes, even though the government announced them as key points of contact for patients, and moreover politicians made promises that could not be fulfilled by the local GPs (20). An internet survey of pediatric practice owners and assistant health personnel from practices in Saarland, Germany confirmed, in keeping with the findings of our study, that pediatricians were able to retrieve sufficient information, but mentioned that cooperation with health authorities proved difficult at times (21).

A survey of pediatric clinicians in Israel supports our qualitative finding that an extensive use of face masks was a challenge for pediatric health professionals: Of 356 respondents in the study, the majority (82%) reported, that mask-wearing interrupted their ability to interact with children and that the children were more fearful (63%). The study also indicated that clinicians with more experience reported that, although things did get more difficult, the change was not as pronounced. As it is more common to wear masks in clinical encounters than in outpatient care, the difficulties might be greater in outpatient care (22). Similarly, the pediatricians in our sample reported that examining children was hampered significantly by the wearing of a mask. However, the lack of PPE was rarely mentioned by the pediatricians in our sample, whereas this was a greater problem for GPs, as the study on Germany's GPs during the pandemic concluded (20).

Other studies also describe a general decline in patient numbers in pediatric practices (15, 26, 46, 47). This differs slightly from the findings in our study and another study on pediatric primary care in Germany during the early COVID-19 pandemic, where almost all pediatricians stated that they were able to catch up with regular health check-ups (27). They saw the decline in patient numbers as being more strongly associated with the absence of acute infections due to social distancing and infection protection measures.

A study involving all 28 Italian pediatric scientific societies showed that the pediatricians had a positive view of telemedicine, especially for chronically ill patients. However, they also agreed that telemedicine could not replace in-person visits for acute patients (47). This supports our results, since the pediatricians in our sample described telemedicine as sometimes helpful, yet felt that face-to-face contact was essential for examining patients with acute conditions, especially small children who cannot verbalize their distress. In a qualitative study on the impact of the pandemic on pediatric health services in North of Scotland and North of England, pediatricians described rapid changes in communication with patients using new technologies, but also within the professional community. Participants in the study described improved collaboration between colleagues in different locations, which indicates that support for telemedicine is likely to grow in the future (48). However, the healthcare context in this study is specific and rather unique because generalist and pediatric services have to provide care in sparsely populated regions with underdeveloped infrastructure in the North of Scotland and England, which includes some remote islands. Thus, the context of these experiences differs from the context of our study.

Alternative ways of providing consultation were also described in a qualitative study on outpatient care in rural areas of Germany (45). Telephone consultation was increased, as well as home visits and creative solutions such as consultations through windows, which was also described by some of our study participants, e.g., performing COVID-19 swabs and examining children in carparks.

In a narrative review of the impact of the pandemic on routine childhood vaccinations, it was concluded that there was a decline in vaccination coverage in many countries (28). In contrast, the pediatricians in our sample stated an overall decline in patient visits, but all of them reported that they managed to catch up with most preventive examinations. Vaccinations sometimes proved to be even easier to be administered on time as children suffered less from acute infections, which can otherwise cause a delay to immunization.

## Strengths and limitations

To the best of our knowledge, this is the first qualitative study with the aim of understanding the experience of the COVID-19 pandemic among German pediatricians. A strength of this study is that we succeeded in conducting detailed qualitative interviews with pediatricians, a target group that was hard to reach during the pandemic due to an increased workload. Our results cannot be generalized to all pediatricians. However, qualitative studies do not claim to generate statistically but theoretically representative data (34). Our interviews enabled us to gain an in-depth understanding of the daily working life of pediatricians during a pandemic. The interviewers (EMG and JvS) are experienced qualitative researchers who have conducted various interviews with different target groups on different topics and were thus able to ensure the professional conduct of the interviews and a rich outcome. All researchers involved in this study, have a great interest in health literacy, health care and health systems; none of them was a pediatrician or physician. Especially when conducting the interviews, this may have been an advantage, as the interviewed pediatricians elaborated more in detail on their daily work: they assumed that practice organization and procedures were unknown to the interviewers. JvS at the time of the interviews was mother of a child in kindergarten and a child in primary school, JC who supported the analysis of the data was at the time mother of a child in kindergarten. Thus, both were up-to date on the situation within those care institutions and affected in their work by the lack of childcare. This might have led to extended discussions on non-medical topics during the interviews as well as on putting a focus on those topics during the analysis. In conclusion, the interest in shedding light on pediatricians’ experiences during the COVID-19 pandemic was professional and personal.

We were able to recruit a broad sample of pediatricians in terms of location of practice (rural vs. urban), region (Bavaria/North-Rhine Westphalia), age and experience and different practice set-ups (individual vs. shared) and could thus capture a wide variety of different perspectives. We assume there are differences, e.g., in regard to socio-economic and ethnic backgrounds of families, depending on location and region of the practice. However, we did not collect data on the patients.

It might be considered a limitation that interviews were conducted via telephone. Observing facial expressions, gestures, or surroundings was not possible. However, recruitment was significantly facilitated by offering telephone interviews as interviews could be scheduled flexibly and there were no inhibiting technical requirements for the participants.

Another limitation of this study is the rapidly changing situation regarding COVID-19. The interviews were conducted from July 2020 to February 2021. During this time, changes occurred quickly, and some impressions and experiences might therefore have changed or developed in different ways after the interviews. This study can only provide insight into a certain time span during the pandemic, however, interviewing over the course of 8 months provides richer insights into the variety of challenges faced by pediatricians over time. Moreover, the study was conducted in Germany and can thus not be transferred to other countries as the health systems are set up differently and the pandemic was handled differently in other countries.

## Conclusion

To our knowledge, this is the first qualitative study focusing in-depth on pediatricians’ experiences during the COVID-19 pandemic. By understanding the challenges and how pediatricians dealt with them, comprehensive pediatric care may be improved in “normal” times, as well as in future pandemic situations.

Pediatricians had to deal with various challenges in providing patient consultation and in organizing care throughout the pandemic. However, the study participants were committed to offering comprehensive support for children and families by providing information on health-related questions as well as on regulatory issues at all times. Positive experiences with reorganizing pediatric practices should be disseminated as “best practices” in order to improve future pediatric health services. Future research could explore whether some of the positive experiences in reorganizing care during the pandemic might be maintained by the pediatricians in the future, such as the separation of cases of acute infections and preventive appointments as well as the continuation of telephone and video consultations in some cases.

## Data Availability

The data presented in this study are available on request from the corresponding author. The data are not publicly available due to the need to preserve the anonymity of the interview partners.
