# Supplementary material for: Pediatricians’ experiences of managing outpatient care during the COVID-19 pandemic: A qualitative study in Germany
Source: Front Pediatr. 2023 Apr 17;11:1127238. doi: 10.3389/fped.2023.1127238 (PMC10150929; doi:10.3389/fped.2023.1127238)
Supplement: Supplementary file 1 [file Datasheet1.docx]

**Appendix 1: Interview Guide**

1. How did your daily work change **due to COVID-19**?
   1. How did the consultations change? Do you have the impression that parents are/have been uncertain? Could you name some examples?
   2. Did your patients continue to attend preventive medical examinations?
      1. Who continued to attend preventive medical examinations? Who did not?
      2. If not, or less: why not?
      3. If not, how did you follow up on missed appointments? (telephone call, sending a request, etc.)
      4. Will the routine clinical examinations be caught up on?
2. How are you handling information about COVID-19? How well do you feel informed (very well, well, not so well, not well at all)? Why?
3. Which information sources on COVID-19 do you use?
4. How do you handle inconsistent (unstable or changing) information?
5. How do you implement the **COVID-19 measures** relevant to you and your work and how do you communicate this to others (patients/colleagues)?
6. What previous knowledge or **lay conceptions** do parents bring to consultations? (*fears, myths, personal experience, Google, social media…etc.*)
   1. To what extent do you feel like some parents are misinformed about COVID-19? How do you notice that?
      1. In your opinion, what are the reasons for patients being misinformed?
      2. How do you deal with misinformed patients? Could you give some **examples** to this **from your everyday care**?

*The interviews continued with questions on health literacy and early childhood allergy prevention.*
